# Depressed heart rate variability predicts adverse neonatal events and outcomes, with important caveats

**DOI:** 10.1038/s41390-026-04897-6

**Published:** 2026-03-10

**Authors:** Karen D. Fairchild

**Affiliations:** Department of Pediatrics, University of Virginia School of Medicine, Charlottesville, VA USA

Heart rate variability (HRV) is an important indicator of health, with frequent small accelerations and decelerations mediated by the autonomic nervous system. With sympathetic activation, norepinephrine release at the sinoatrial node of the heart leads to an increase in heart rate, whereas with parasympathetic activation, acetylcholine is released, which slows the heart rate. Various pathologic conditions lead to depressed HRV. In adults, for example, severe diabetes, chronic kidney disease and heart failure are associated with low HRV, which portends adverse outcomes. In the fetus, an ominous heart rate pattern of low variability and superimposed decelerations can indicate compromised perfusion and oxygenation. The fetal inflammatory response associated with chorioamnionitis and the systemic inflammatory response in neonatal sepsis also lead to decreased HRV.^1^ Various types of neonatal brain injury, including acute hypoxic-ischemic^2^ or hemorrhagic injury,^3^ can lead to abnormal heart rate patterns and can predict adverse neurodevelopmental outcomes.^4^ Using HRV to predict future adverse events and outcomes (particularly actionable ones) is an important area of investigation.

In extremely preterm neonates, whether and how abnormal HRV predicts future intraventricular hemorrhage (IVH) rather than simply reflecting injury that has already occurred is a matter of ongoing research. In this issue of Pediatric Research, Smolkova and colleagues make progress on this front, reporting on HRV in the first week after birth in relation to the occurrence and timing of severe (grade 3–4) IVH in 48 infants <29 weeks gestation, 7 of whom had sIVH.^5^ As background for this work, the group recently published a scoping review in which six of nine studies investigating HRV and IVH reported an association between the two, with three of four studies finding that low HRV preceded recognition of IVH on ultrasound.^6^ In the current work, the important finding is that, of the eight metrics studied, the two with the strongest association with sIVH, SD1 and HF, reflect depressed parasympathetic function. Of note, the ten hours of electrocardiogram data analyzed were selected as five 2-h segments free of artifact obtained in the first week. Precise timing of sIVH in relation to the HRV findings is difficult to ascertain, but they report that 5 infants had a pattern of low HRV before sIVH was recognized on ultrasound.

Autonomic nervous system maturation in the fetus and the preterm-born neonate proceeds from sympathetic to parasympathetic.^7^ The latter is important in the regulation of cerebral blood flow, and it is biologically plausible that immature parasympathetic function predisposes to IVH. Measuring HRV as an indicator of autonomic function in neonates is complex, and this group used multiple methods, 4 linear and 4 non-linear, accounting for both stationary and non-stationary patterns. With linear methods, consecutive electrocardiogram QRS complexes are analyzed with either time series (e.g., standard deviation) or with power spectral analysis generally involving Fourier transform of the time series followed by analysis of power in low and high frequency (HF) time ranges. HF-HRV, if measured properly, can assess parasympathetic function, particularly respiratory sinus arrhythmia in which HR increases with inhalation and decreases with exhalation. The ideal way to measure this is with a consistent and relatively slow respiratory rate; in adults, the frequency range to measure HF-HRV is 0.15–0.4 Hz (9–24 breaths per minute), and paced breathing is used when feasible. Smolkova and colleagues used a wide range of 0.2–2 Hz corresponding to 12–120 breaths per minute. Of note, when the respiratory rate is more than half the heart rate, cardiac aliasing can occur^8^ based on the Nyquist theorem, making HF signals appear as low frequency (as when a spinning wheel appears to go backward in a video). This calls into question the value of frequency-based HRV analysis, especially for infants on invasive mechanical (sometimes high-frequency) ventilation. Appropriately, the Smolkova group additionally reports on four non-linear metrics (two Poincare and two detrended fluctuation analysis) to account for the non-stationary nature of HR patterns in the 10-h period of analysis. In total, they found that 7 of the 8 HRV metrics were lower in infants with sIVH, and two specific for parasympathetic function were particularly associated with sIVH not yet detected on ultrasound.

In addition to reproducing these results in a larger, multicenter cohort, a number of challenges remain before routine implementation of heart rate pattern analysis to predict IVH and other adverse events and outcomes in Neonatal Intensive Care Unit (NICU)  patients. Medications known to impact HRV should be taken into account; for example, atropine depresses HRV through anticholinergic action, and steroids increase HRV at least in part by reducing inflammation.^9^ The impact of caffeine on preterm infant HRV has not been established. Other indicators of cardiorespiratory instability, such as swings in blood pressure and blood carbon dioxide content, can impact cerebral perfusion and IVH risk and may alter heart rate patterns. Another major challenge is determining timing of IVH. Even with daily head ultrasounds, it is not possible to precisely determine when brain hemorrhage starts. And finally, more work is needed to determine whether the finding of abnormal HRV can lead to interventions to decrease occurrence or extension of IVH. Many centers have implemented “IVH bundles” to minimize swings in cerebral blood flow, and this has reduced severe IVH rates in some but not all reports.^10^ Indomethacin prophylaxis has been shown in randomized clinical trials to reduce sIVH but many clinicians have opted against routine use due to adverse effects and uncertain long-term benefit. The El Dib group recently published their experience implementing a clinical practice guideline for prophylactic indomethacin from 2016 on, and did not find that sIVH was reduced in a before/after study.^11^ The date range of the 48 infants included in this HRV project is not stated, but presumably some received indomethacin which has vasoconstrictive and anti-inflammatory effects but unknown impact on HRV.

With the increasing use of AI in medicine, it is likely that in the next decade or two we will have the ability to access vital sign-based scores indicating risk of various adverse events and outcomes. Combining analysis of heart rate patterns, blood pressure, blood gases, and cerebral oxygenation with known clinical risks is likely to improve identification of impending or existing IVH. Vital sign analysis may also be useful for predicting adverse neurodevelopmental outcomes and identify a subset of patients for whom earlier targeted therapies may improve long-term outcomes. Physiologic biomarkers including HRV could also serve as research tools for risk stratification in clinical trials. Of critical importance, as we consider whether and how to implement predictive algorithms in the NICU, is assuring that any additional cost, clinician work, patient testing, and parent worry is outweighed by improvement in patient outcomes.
